# How Do Scholars Conceptualize and Conduct Health and Digital Health Literacy Research? Survey of Federally Funded Scholars

**DOI:** 10.2196/57040

**Published:** 2024-10-31

**Authors:** Mayank Sakhuja, Brooks Yelton, Simone Kavarana, Lauren Schaurer, Jancham Rachel Rumthao, Samuel Noblet, Michelle A Arent, Mark M Macauda, Lorie Donelle, Daniela B Friedman

**Affiliations:** 1 UNC Lineberger Comprehensive Cancer Center University of North Carolina at Chapel Hill Chapel Hill, NC United States; 2 Gillings School of Global Public Health University of North Carolina at Chapel Hill Chapel Hill, NC United States; 3 Department of Health Promotion, Education, and Behavior Arnold School of Public Health University of South Carolina Columbia, SC United States; 4 Envera Health Richmond, VA United States; 5 Department of Athletics University of South Carolina Columbia, SC United States; 6 Center for Applied Research and Evaluation Arnold School of Public Health University of South Carolina Columbia, SC United States; 7 Department of Biobehavioral Health and Nursing Science College of Nursing University of South Carolina Columbia, SC United States

**Keywords:** health literacy, digital health literacy, eHealth literacy, social determinants of health, SDoH, research scholarship, health care, public health research, digital health tools, community health

## Abstract

**Background:**

The concept of health literacy (HL) is constantly evolving, and social determinants of health (SDoH) have been receiving considerable attention in public health scholarship. Since a 1-size-fits-all approach for HL fails to account for multiple contextual factors and as a result poses challenges in improving literacy levels, there is a need to develop a deeper understanding of the current state of HL and digital health literacy (DHL) research.

**Objective:**

This study examined scholars’ conceptualization and scope of work focused on HL and DHL.

**Methods:**

Using a search string, investigators (N=2042) focusing on HL, DHL, or both were identified from the grantee websites of the National Institutes of Health RePORTER (RePORT Expenditures and Results) and the Canadian Institutes of Health Research. The investigators were emailed a survey via Qualtrics. Survey questions examined the focus of work; whether the investigators studied HL/DHL in combination with other SDoH; the frameworks, definitions, and approaches used; and research settings. We analyzed survey data using SPSS Statistics version 28 and descriptive analysis, including frequencies and percentages, was conducted. Chi-square tests were performed to explore the association between the focus of work, settings, and age groups included in the investigators’ research.

**Results:**

A total of 193 (9.5%) of 2042 investigators responded to the online survey. Most investigators (76/153, 49.7%) were from public health, 83/193 (43%) reported their research focused on HL alone, 46/193 (23.8%) mentioned DHL, and 64/193 (33.2%) mentioned both. The majority (133/153, 86.9%) studied HL/DHL in combination with other SDoH, 106/135 (78.5%) conducted HL/DHL work in a community setting, and 100/156 (64.1%) reported not using any specific definition to guide their work. Digital tools (89/135, 65.9%), plain-language materials (82/135, 60.7%), and visual guides (56/135, 41.5%) were the top 3 approaches used. Most worked with adults (131/139, 94.2%) and all races and ethnicities (47/121, 38.8%).

**Conclusions:**

HL and DHL research largely considered SDoH. Multiple HL tools and approaches were used that support the examination and improvement of literacy and communication surrounding health care issues.

## Introduction

### Background

The concept of health literacy (HL) is constantly evolving. The Healthy People 2030 initiative defines HL as “the degree to which individuals have the ability to find, understand and use information and services to inform health-related decisions and actions for themselves and others.” [1]. It refers to individuals’ using their cognitive and social skills for gaining access to, understanding, and using information for promoting and maintaining good health for themselves and for others around them [2]. According to the United Nations Educational, Scientific and Cultural Institution, about 388 million adults in South and Southwest Asian countries continue to grapple with illiteracy, coupled with a lack of basic HL [3]. Even though many high-income nations, such as the United States, Canada, the United Kingdom, and Australia, have included HL in their national agenda [4], more than one-third of individuals in the United States still have limited HL, which consequently results in misunderstanding of medical information, increased health care–related costs, and poor health outcomes [5,6]. Individuals who face difficulty in understanding health-related materials may experience shame and discomfort in interacting or sharing their medical concerns and questions with health professionals [7].

### Health Literacy as a Social Determinant of Health

HL functions as a critical component within the context of other social determinants of health (SDoH), such as education, socioeconomic status, access to quality health care, neighborhood and built environments, and social support networks [8,9]. Low literacy levels impact an individual’s ability to access and use health care services by making it challenging for them to navigate health systems, understand health insurance information, and adhere to treatment plans resulting in delayed or inadequate care [10,11]. Individuals with a low socioeconomic status often have low HL levels, leading to difficulties in understanding providers’ instructions, managing illness, and making informed health care choices for themselves [12]. Although studies indicate education may be associated with HL, the data cannot speak to a direct cause and effect tying higher education levels to higher HL [13]. Education is not a reliable proxy for determining literacy, as individuals with higher education may still have difficulty in understanding information pertaining to their health or the health of their families.

### Navigating Health Care and Health Information

HL plays an important role in reducing health disparities, empowering individuals, and improving patient-provider communication and health outcomes. Individuals who possess high levels of HL are in a better position to understand medical instructions provided by their doctors, actively participate in decisions related to their health, and navigate the complex health care system. Changes within the health care environment, specifically the increased use of digital communication platforms, have transformed health care delivery and highlighted additional concerns around optimal patient-provider engagement. Digital health literacy (DHL) has become a significant component of HL with the increasing use of digital technologies in health care, including telehealth platforms, wearable devices, and mobile health apps and websites [14]. Although DHL shares core conceptual elements of HL, it differs as it requires an individual to possess additional skills to use technological devices, such as mobile phones and computers, and skills to use those devices to search and evaluate health-related information available online through multiple sources [14]. It is therefore essential for individuals to possess DHL skills to navigate digital technologies, access accurate and reliable online health information, and engage with telehealth platforms.

### New Challenges and Directions

Despite the recognition of HL and DHL as essential factors of health care quality and outcomes, challenges exist in achieving optimal levels of literacy skills nationally and internationally [15]. Additionally, the increasing focus on DHL interventions poses new challenges in improving DHL, such as possessing skills to use new digital health technologies and identifying credible sources of information online [16]. Furthermore, a 1-size-fits-all approach for HL and DHL fails to account for cultural and linguistic diversity, age and generational differences, multiple health conditions and contexts, unequal access to digital resources, and diverse HL levels [17]. To develop a deeper understanding of the current state of HL and DHL research, we reported the results of a survey of HL and DHL researchers in this study. The aim was to comprehensively examine researchers’ conceptualization of and approaches to HL and DHL work, identify strategies, and highlight areas for further investigation and intervention. This research addressed the following research questions:

- What concepts, definitions, and frameworks guide researchers’ HL and DHL research?
- What specific settings and methods are used by HL and DHL researchers?
- Which SDoH are addressed in HL and DHL research?
- Who are the participants in HL and DHL research?

## Methods

### Survey Development and Measures

We used an iterative approach to develop the survey as per CHERRIES (Checklist for Reporting Results of Internet E-Surveys) [18]. Two team members drafted the initial list of questions, and all team members, including experts in HL and DHL, weighed in on multiple drafts. To ensure the survey’s reliability, we consulted content experts to design survey questions and response options that were appropriately worded and meaningful to study participants. Since we did not aim to measure any specific constructs, we did not conduct the type of survey validation that we would have done otherwise. Survey questions examined researchers’ specific focus (HL, DHL, or both); funding sources for HL and DHL research; and definitions, conceptual frameworks, and approaches used to study HL and DHL. We were also interested in understanding whether respondents studied HL/DHL in combination with other SDoH, as well as the research settings and geographic locations of their research. We asked about their main discipline/affiliation, number of years studying HL or DHL, educational qualification, and place of employment. Finally, we inquired about the data collection methods used in their research, whether they had ever received mentorship in HL/DHL work, or whether they provided mentorship to others. Respondents could select more than 1 response option for several of the survey questions.

### Ethics Approval

This study was considered exempt by the University of South Carolina (USC) Office of Research Compliance under approval number Pro00124306.

### Participant Recruitment and Survey Dissemination

We applied a search string (“literacy” or “health literacy” or “digital health literacy” or “e literacy” or “e health” or “m health” or “e health literacy”) to project titles, project terms, and project abstracts on the grantee website of the National Institutes of Health (NIH) RePORTER (RePORT Expenditures and Results) and the Canadian Institutes of Health Research in November 2022. Project investigators (PIs) of studies matching those parameters were included in the sample. We obtained the email addresses of the PIs directly from the NIH RePORTER website and manually searched for email addresses of the PIs selected from the Canadian Institutes of Health Research grantee website. We excluded PIs for whom email addresses were unavailable on the NIH RePORTER grantee website, as well as PIs from the Canadian Institutes of Health Research whose email addresses could not be located through manual searches. The final sample included 2042 individual investigators. We also distributed the survey link via 2 organizational listservs consisting of members focused on health communication and health promotion research and practice.

We programmed the survey questions in Qualtrics [19]. Next, we prepared separate email lists for NIH and Canadian Institutes of Health Research PIs in Qualtrics, and invitations to participate in the survey were sent on January 23, 2023. The invitation email included a brief description and purpose of the study and a link to the Qualtrics survey and informed participants that survey completion would take approximately 15-20 minutes. Five email reminders were sent from Qualtrics at regular intervals to encourage participation and completion of the survey before its scheduled closure on April 23, 2023. All survey respondents were entered into a random drawing for 1 of 5 US $25 Amazon gift cards.

### Data Analysis

Data were downloaded into Microsoft Excel. IBM SPSS Statistics version 28 [20] was used to conduct descriptive analysis including frequencies and percentages, and chi-square tests with a significance level of *P*<.05 were performed to explore the association between respondents’ research focus (HL, DHL, both) and the settings and age groups with whom they conducted their research. We included responses from all 193 respondents and did not eliminate surveys that had missing responses. Analysis was conducted in a way that the results for each survey question contained only the valid responses for that question with the relevant denominator.

## Results

### Survey Respondent Characteristics

A total of 193 respondents answered the survey questions. Almost half (76/153, 49.7%) reported public health as their main discipline, 41/153 (26.8%) reported medicine, 38/153 (24.8%) reported health services/policy, 21/153 (13.7%) were from education, and 17/153 (11.1%) identified nursing as their academic discipline. Most had a PhD (98/132, 74.2%), 16/132 (12.1%) were MDs, and 13/132 (9.8%) had a master’s degree as their highest educational qualification. Employment settings ranged from university (93/132, 70.5%), hospital system (12/132, 9.1%), or research institution (10/132, 7.6%). Most (106/132, 80.3%) of the respondents had never received any formal mentorship in HL or DHL. Those who had received formal mentorship reported receiving training from an academic mentor, professional organization seminars, or professional training. Just over half (73/142, 51.4%) reported that they had been studying HL or DHL for 1-5 years, and 37/142 (26.1%) indicated more than 10 years of experience studying HL or DHL.

### Focus of Research

Regarding the scope of work, 83 (43%) of 193 respondents reported that most of their work focused on HL, while 46 (23.8%) reported DHL, and the remaining 64 (33.2%) indicated a dual focus including both HL and DHL. When asked about the specific topics of focus, 87/139 (62.6%) respondents reported that they had a general focus on HL/health communication, 61/139 (43.9%) focused on topics around social justice/health equity, 48/139 (34.5%) focused on face-to-face patient-provider communication, 41/139 (29.5%) focused on misinformation/disinformation, 37/139 (26.6%) focused on virtual patient-provider communication, 36/139 (25.9%) focused on peer-to-peer health conversations, and 27/139 (19.4%) focused on policy development. Respondents had almost equal focus on specific health behaviors, including healthful eating (36/139, 25.9%), physical activity (36/139, 25.9%), and cancer screening (33/139, 23.7%).

### Concepts and Frameworks Used in HL or DHL Research

About two-thirds (100/156, 64.1%) of the respondents reported not using any specific definition of HL or DHL to guide their work. Of those who did follow a specific definition (n=56, 35.9%), roughly 19 (33.9%) indicated using the Institute of Medicine’s [21] definition. Other definitions cited were by Nutbeam [22], the World Health Organization [23], U.S. Department of Health and Human Services [1,24], among others, as described in Table 1. Research by about one-third (46/143, 32.2%) of the respondents was guided by the Healthy People 2030 framework [1]; 35/143 (24.5%) used the Nutbeam framework that describes HL as functional, interactive, and critical [22]; 27/143 (18.9%) used the HL skills framework by Squiers et al [25]; and 18/143 (12.6%) used the HL systems capacity framework by Sorenson et al [26]. Regarding DHL research, 17/143 (11.9%) respondents used the electronic health literacy (eHL) framework by Bautista [27], 12/143 (8.4%) used the eHL definition and the Lily model by Norman and Skinner [14], 12/143 (8.4%) used the eHL framework by Norgaard et al [28], and 10/143 (7%) used the consumer eHL taxonomy by Chan et al [29]. Some respondents (6/143, 4.2%) did not follow any framework, whereas others mentioned they used dissemination and implementation science–based, collaborator-informed socioecological models and environmental HL frameworks. Frameworks used in HL and DHL research are presented in Table 2.

**Table 1:** Definitions used in HL^a^ and DHL^b^ research provided by respondents.

Definition guiding HL and DHL research	Respondents (n=56), n (%)	Source
The individuals’ capacity to obtain, process, and understand basic health information and services needed to make appropriate health decisions.	19 (33.9)	Institute of Medicine [21]
HL represents the personal knowledge and competencies that accumulate through daily activities and social interactions across generations. Personal knowledge and competencies are mediated by organizational structures and availability of resources that enable people to access, understand, appraise, and use information and services in ways that promote and maintain good health and well-being for themselves and those around them.	3 (5.4)	World Health Organization [23]
The degree to which individuals have the capacity to obtain, process, and understand basic health information and services needed to make appropriate health decisions.	3 (5.4)	U.S. Department of Health and Human Services [24]
Personal HL is the degree to which individuals have the ability to find, understand, and use information and services to inform health-related decisions and actions for themselves and others. Organizational HL is the degree to which organizations equitably enable individuals to find, understand, and use information and services to inform health-related decisions and actions for themselves and others.	3 (5.4)	U.S. Department of Health and Human Services [1]
The degree to which individuals and groups can obtain process, understand, evaluate, and act upon information needed to make public health decisions that benefit the community.	3 (5.4)	Freedman et al [30]
The personal, cognitive, and social skills that determine the ability of individuals to gain access to, understand, and use information to promote and maintain good health.	2 (3.6)	Nutbeam [22]
The cognitive and social skills that determine the motivation and ability of individuals to gain access to, understand, and use information in ways that promote and maintain good health.	2 (3.6)	Nutbeam [31]
The knowledge, skills, and abilities that pertain to interactions with the health care system.	2 (3.6)	Ishikawa and Yano [32]
The ability to seek, find, understand, and appraise health information from electronic sources and apply the knowledge gained to addressing or solving a health problem.	2 (3.6)	Norman and Skinner [14]
DHL involves the context and ability to use digital technologies to access, understand, and use health information in a timely manner to improve health outcomes.	1 (1.8)	Norman and Skinner [14]
DHL is defined as the ability to appraise health information from electronic sources and apply the knowledge gained to address or solve a health-related problem and as such has emerged as an important component of greater HL. Although DHL shares core aspects of HL, DHL is distinguished by additional skills: computer literacy, the ability to use computers and related technology efficiently to accomplish tasks, media literacy to use search engines, and information literacy to evaluate a wide variety of sources.	1 (1.8)	Smith and Magnani [16]
The ability to understand and interpret the meaning of health information in written, spoken, or digital form and how this motivates people to embrace or disregard actions relating to health.	1 (1.8)	Adams et al [33]
The capacity to obtain, interpret, and understand basic health information and services and the competence to use such information to enhance health.	1 (1.8)	Pavlekovic [34]
At its most basic, environmental HL has been described as an ability to make connections between environmental exposures and human health. Representations of environmental HL tend to start with individual understanding of specific risks and then lead to broader understanding, including strategies that empower people to reduce or eliminate environmental exposures that can harm health.	1 (1.8)	Finn and O’Fallon [35]
HL is defined as the ability to access, understand, and use information to make health decisions. It is well known that low HL is associated with poorer health outcomes.	1 (1.8)	Berkman et al [36]
The knowledge and skills required to understand and use information relating to health issues, such as drugs and alcohol, disease prevention and treatment, safety, accident prevention, first aid, emergencies, and staying healthy.	1 (1.8)	Australian Bureau of Statistics [37]
Knowledge and beliefs about mental disorders that aid their recognition, management, or prevention. It is a multidimensional concept that includes (1) an ability to recognize specific disorders or types of psychological distress, (2) knowledge and beliefs about risk factors and prevention, (3) knowledge and attitudes that facilitate help seeking, and (4) knowledge and beliefs regarding formal and informal intervention approaches. DHL focuses more on knowledge and skills related to technology-based approaches to digital health.	1 (1.8)	Jorm [38]
The ability to access, understand, evaluate, and communicate information as a way to promote, maintain, and improve health in a variety of settings across the life course.	1 (1.8)	Rootman and Gordon-El-Bihbety [39]
The ability to make sound health decision(s) in the context of everyday life: at home, in the community, at the workplace, in the health care system, in the marketplace and in the political arena. It is a critical empowerment strategy to increase people’s control over their health, their ability to seek out information, and their ability to take responsibility.	1 (1.8)	Kickbusch et al [40]
The wide range of skills and competencies that people develop to seek out, comprehend, evaluate, and use health information and concepts to make informed choices, reduce health risks, and increase the quality of life.	1 (1.8)	Zarcadoolas et al [41]
An emerging field in the intersection of medical informatics, public health, and business, referring to health services and information delivered or enhanced through the internet and related technologies. It is also a state of mind, a way of thinking, an attitude, and a commitment for networked, global thinking, to improve health care locally, regionally, and worldwide by using information and communication technology.	1 (1.8)	Eysenbach [42]
We define mental HL as the extent to which individuals are knowledgeable about mental health problems and their treatment.	1 (1.8)	N/A^c^
Individuals’ ability to apply for/maintain health coverage and navigate the health care system to have their health needs met.	1 (1.8)	N/A
Functional HL is a person’s ability to comprehend patient education materials to make an informed decision about their health.	1 (1.8)	N/A
We used an internal definition (unpublished) focused on literacy related to health, research, medicine, and insurance to guide our work.	1 (1.8)	N/A

^a^HL: health literacy.

^b^DHL: digital health literacy.

^c^N/A: not applicable.

**Table 2:** Frameworks used in HL^a^ and DHL^b^ research.

Conceptual framework guiding HL and DHL research	Respondents (n=143), n (%)^c^
Healthy People 2030 (U.S. Department of Health and Human Services [1])	46 (32.2)
HL as functional, interactive, and critical (Nutbeam [22])	35 (24.5)
HL skills framework (Squiers et al [25])	27 (18.9)
HL systems capacity (Sorenson et al [26])	18 (12.6)
eHL (Bautista [27])	17 (11.9)
HL as an asset model (Nutbeam [43])	15 (10.5)
eHL and Lily model (Norman and Skinner [14])	12 (8.4)
eHL framework (Norgaard et al [28])	12 (8.4)
Consumer eHL (Chan et al [29])	10 (7.0)
eHL framework (Kayser et al [44])	8 (5.6)
Integrated model for cancer screening (Best et al [45])	7 (4.9)
Other frameworks	34 (23.8)
None	6 (4.2)

^a^HL: health literacy.

^b^DHL: digital health literacy.

^c^Participants could select more than 1 response option, so the total percentage may be more than 100%.

### Methods and Settings Used in HL or DHL Research

Table 3 presents the settings in which respondents conducted their research. Most conducted their HL/DHL work in a community setting (106/135, 78.5%); 49/135 (36.3%) reported a primary care setting; 44/135 (32.6%) reported a specialty care setting; 29/135 (21.5%) reported the virtual health care space, such as patient portals/provider portals; and 20/135 (14.8%) reported government-/policy-level settings. Chi-square tests indicated that of those (n=20, 14.8%) who conducted research in a government-/policy-level setting, 14 (70%) were focused on HL research, while 13 (44.8%) of the respondents who conducted research in the virtual health care space were focused on DHL. Only significant (*P*<.05) chi-square test results are presented in Table 4. When asked about the specific approaches that they used in their work, 89 (65.9%) respondents mentioned using digital tools, such as apps, websites, patient portals, virtual visits, and wearable devices; 82/135 (60.7%) reported using plain-language materials; 56/135 (41.5%) used visual guides, such as graphical decision tools; 39/135 (28.9%) used teach-back strategies; and 36/135 (26.7%) made use of a partnership with patient navigators. Most respondents used mixed methods (109/134, 81.3%) for their work. For data collection, 112/134 (83.6%) respondents reported that they collected data using interviews, 118/134 (88.1%) conducted surveys, 89/134 (66.4%) conducted focus groups, 50/134 (37.3%) conducted secondary data analysis, and 44/134 (32.8%) completed scoping and systematic reviews and meta-analysis. Other types of data collection methods included content analysis (33/134, 24.6%), comprehension testing (23/134, 17.2%), readability testing (25/134, 18.7%), and observational data (42/134, 31.3%).

**Table 3:** Settings in which HL^a^ and DHL^b^ research is conducted.

Research settings			Respondents (n=135), n (%)^c^
Clinical setting			108 (80.0)
	Primary care	49 (36.3)	
	Specialty care	44 (32.6)	
	Other	15 (11.1)	
Community setting			106 (78.5)
Government-/policy-level setting			20 (14.8)
Virtual health care space^d^			29 (21.5)
Other settings^e^			16 (11.9)
	Academia	7 (5.2)	
	County hospital	1 (0.7)	
	Higher education level	1 (0.7)	
	Hospital	1 (0.7)	
	Interactive internet to classrooms	1 (0.7)	
	Medical center	1 (0.7)	
	Mobile health	1 (0.7)	
	School	1 (0.7)	
	Smoking quitline	1 (0.7)	
	Tribal health care organization	1 (0.7)	

^a^HL: health literacy.

^b^DHL: digital health literacy.

^c^Participants could select more than 1 response option, so the total percentage may be more than 100%.

^d^Patient or provider portals.

^e^Respondent write-in.

**Table 4:** Chi-square tests between focus of research and research setting.

Focus	Government-/policy-level setting (n=20; *P*=.034), n (%)	Virtual health care space (n=29; *P*=.006), n (%)	Specialty care (n=44; *P*=.014), n (%)
DHL^a^	2 (10.0)	13 (44.8)	16 (36.4)
HL^b^	14 (70.0)	6 (20.7)	11 (25.0)
Both	4 (20.0)	10 (34.5)	17 (38.6)

^a^DHL: digital health literacy.

^b^HL: health literacy.

### Consideration of Social Determinants of Health

Most respondents (133/153, 86.9%) reported that they studied HL/DHL in combination with other SDoH. Health care access and quality were studied with HL/DHL most often (93/133, 69.9%), followed by social and community contexts (80/133, 60.2%), education access and quality (57/133, 42.9%), neighborhood and built environment (44/133, 33.1%), and economic stability (40/133, 30.1%). Other SDoH, as reported by respondents, included equity, gender and caregiving support, insurance and employment status, language equity, mental health stigma, poverty, culture, environmental exposures, and other measures of socioeconomic status.

### Profile of Participants With Whom Respondents Conducted Research

Respondents conducted research mostly with adult populations (131/139, 94.2%), 42/139 (30.2%) conducted research with adolescents (12-17 years old), and 23/139 (16.5%) conducted research with youth (<12 years old). There were no significant chi-square test results between the focus of research and the age categories of respondents’ research participants. Some respondents reported conducting research with all/diverse races (47/121, 38.8%), while others indicated a more limited approach. One-third (40/121, 33.1%) respondents conducted research with only Black, African American, or both populations; 31/121 (25.6%) reported research with White/Caucasian/European Americans; 32/121 (26.4%) reported research with Hispanic or LatinX populations; and 11/121 (9.1%) reported research with Asian, South Asian, and East Asian populations. Most respondents (83/137, 60.6%) reported that they conducted research with the general public, 43/137 (31.4%) reported research with general patients, and 58/137 (42.3%) reported research with patients of a specific health/medical specialty. About one-third (44/137, 32.1%) of the respondents conducted research with general clinical providers; 43/137 (31.4%) included physicians, nurse practitioners, and PAs as their research participants; and 27/137 (19.7%) worked with professionals who were from nursing and related clinical/health sciences backgrounds. Respondents also conducted research with specific communities who had a specific culture (33/137, 24.1%); the lesbian, gay, bisexual, transgender, queer, questioning, intersex, asexual, and other (LGBTQIA+) community (15/137, 10.9%); individuals without a shelter or stable housing (15/137, 10.9%); and individuals with disabilities (10/137, 7.3%).

## Discussion

### Principal Findings

This study was a comprehensive examination of researchers’ conceptualization of and strategies for conducting HL and DHL research. Understanding principles and methods that guide the work in this area is critical for advancing the field, pursuing needed interventions in both clinical and community settings, and studying literacy in the context of additional social determinants that influence health behaviors and outcomes.

#### Conceptualizing Health and Digital Health Literacy

Most respondents reported not using any specific definition of HL or DHL to guide their work. Others suggested a variety of definitions and seminal papers from the field. Without the use of common definitions and measures, understanding a discipline will be limited to knowledge obtained from individual cases; however, it may not advance the field holistically so that we can create culturally and linguistically appropriate communication strategies to promote evidence-based programs, interventions, and policies [46,47].

Most respondents reported that their research was generally focused on HL or health communication and on social justice or health equity, demonstrating the importance of studying HL and DHL within the context of other social factors. Over 85% of the respondents investigated HL/DHL in relation to other SDoH. Researchers’ attention to HL/DHL and SDoH is consistent with van Kessel et al [48], who claimed that the development of HL, and increasingly DHL, is a pre-requisite for enhanced health and well-being. Others have also highlighted the growing importance of DHL and contend that DHL and digital inclusion constitute the “super–SDoH,” especially given our widespread reliance on digital tools for health [49].

Life contexts (eg, age, gender, income, employment, education, HL/DHL skills, social support, residential environments, health care access) create conditions for (in)equitable health and well-being [48,50]. Researchers in this study focused predominantly on the intersection of HL/DHL and health service system issues. One-third to half of the researchers addressed HL/DHL skills in relation to education, the neighborhood context, and economic stability. With agreement that HL and DHL constitute fundamental skills in support of equitable health and well-being, our findings suggest important opportunities to enhance research activity for greater insight into the impact of HL/DHL skills at the intersection of culturally diverse groups of individuals, those living in poverty, individuals with poor mental health, individuals without stable housing, individuals with disabilities, and across gender and sexual identities [51].

#### Intended Audiences

Almost 80% of the respondents conducted research in a community setting. Community-engaged HL and DHL research is critical to understanding diverse stakeholders’ needs, preferences, and pre-existing beliefs [52]. Effective education programs, interventions, and health communication materials are tailored and culturally relevant to different audiences, centering community-held values, imagery, and voices for both trust in the message/messenger and adoption of associated content and behaviors [53,54]. Codeveloping research, programs, and communication products with community members ensures stakeholder priorities are fully accounted for and increases member buy-in [55]. Although clinical interventions are necessary to improve health and digital literacy at the organizational level, design and delivery must balance pragmatic approaches to implementation with the utility and receptibility of a diverse patient population [56]. For improved prevention, it is also imperative that researchers meet stakeholders where they are, particularly among medically underserved populations that may only engage with the health care system in emergency situations.

Despite the racial and ethnic variety of respondents’ research audiences, limited research was reportedly conducted with Asian, South Asian, or East Asian populations. This may be due, in part, to perceptions that health outcomes tend to be favorable among individuals of Asian descent in the United States and Canada [57], leading researchers to focus their efforts on communities perceived as being at higher risk for negative health disparities. However, researchers can inadvertently disadvantage population subgroups by aggregating groups of people into broad categories, masking heterogeneity and limiting research capacity, health education, and funding for groups that may have different risk profiles or poorer health outcomes than other groups within the same broad category [58,59]. In addition, researchers using a health equity lens must make deliberate strides to improve research participation among many population groups by centering aims around each population’s interests and goals, repairing and building trust, providing adequate compensation and incentive, and tailoring communication to meet the intended population’s language, source, and channel preferences [60]. Regardless, the tenets of value-based health promotion support the need to codevelop health and DHL with individuals and populations across the health-illness continuum, as HL can improve both knowledge of health risks and trust in health care. A smaller portion of respondents conducted research with communities who shared a specific culture, members of the LGBTQIA+ community, individuals without a shelter or stable housing, and individuals with disabilities. These populations are at higher risk for health care bias and other SDoH [61]; thus, it is imperative that they be engaged and appropriately consulted in the field of HL.

It was not surprising that most of our survey respondents conducted research with adult populations (94.2%), whereas a smaller proportion worked with adolescents (30.2%) and the youth (16.5%). Additionally, none of the respondents mentioned using conceptual frameworks adapted for use with younger age groups. This is likely due to the fact that parental involvement and consent are required in adolescent health care and participation in research or other programs. However, it is important to understand and address health and DHL from a life course perspective, as early life experiences and cumulative disadvantages across the lifespan can lead to accumulated stress and uptake of unhealthy behavior, leading to poor health outcomes in adulthood [62]. The limited focus on HL among lower age groups has remained a significant gap requiring more research on HL instruments and interventions among younger age groups [63]. The limited literature focused on HL among adolescents and the youth impedes our understanding of the needs and preferences of HL and DHL in the context of younger age groups.

### Implications for Health Care Professionals

Approximately three-quarters of the respondents reported public health and medicine as their academic and professional disciplines. Although the nursing profession is proportionately greater than the discipline of public health or medicine, only 11% of the respondents conducting HL research had a nursing background. Worldwide, nurses serve as critical partners in the health care encounter as they provide education and facilitate communication exchange, which can aid patient HL [64]. However, research demonstrates that nurses in the United States cannot identify patient communication behaviors that point toward low HL levels [65]. Engaging nursing professionals in HL and DHL research is important because nurses interact directly with patients, which makes them well positioned to develop and implement strategies to fully understand the needs and challenges of a diverse patient population. More involvement of nursing professionals would help in developing informed and tailored communication strategies for patient populations and help determine the most effective ways for communicating health information with patients of varying levels of literacy. HL research by nursing professionals would help in reducing disparities as understanding the challenges by patients from diverse backgrounds would allow nursing professionals to deliver more equitable health care.

### Limitations

Our survey was distributed primarily via lists of researchers who had NIH funding in the United States or funding through the Canadian Institutes of Health Research; thus, our sample primarily reflects researchers who have been awarded through these funding streams. There are likely others in the field who have carried out their work using other funding streams. Since research agendas are, to some extent, directed by the priorities of funding agencies, this survey may have missed individuals conducting different kinds of HL work. Additionally, since we primarily searched the grantee websites of the NIH and the Canadian Institutes of Health Research, our sample of researchers was primarily based in high-income countries. As we could not reach out to researchers in low- and middle-income countries, our study findings cannot be generalized to other countries. Despite keeping the survey open for a period of 3 months and sending 5 reminder emails for completing the survey, the low response rate of our survey further restricts the generalizability of our study findings. As we did not conduct cognitive testing of our survey questions, there is a possibility that our questions did not address the most meaningful information. Finally, the majority of our questions were closed ended, which may fail to capture the complexity of the working definitions used by respondents, as well as the variety of work they engage in. Even though this study has limitations, we strongly believe that the study findings still provide important insights into the scope of and how researchers conceptualize HL and DHL research, which can inform future research directions. Understanding what funded researchers are doing in this area of research and with the growth in the need for research on SDoH, this study can help inform more comprehensive examination of this field of research.

### Conclusion

The results of our survey indicate there is significant variation in how HL is researched and understood. Although a variety of approaches may add richness to the field, it also makes comparisons across studies and generalizability of findings more difficult. Further, few respondents reported receiving any formal mentorship in their study of HL or DHL, which may perpetuate inconsistencies in how HL is defined, since knowledge transfer from one generation of researchers to another may be limited. It is encouraging that the majority of survey respondents investigated HL along with other SDoH, given the inequality that is often pervasive within health systems and the importance of going beyond studies of readability to more contextual factors. However, the lack of work in the clinical setting is problematic, since it has been recognized that HL is a structural issue that the health sector must address. Given the somewhat limited consistency in how the concept is currently defined and the evolution of the field from the study of readability of documents to the exploration of literacy within the context of social factors and health equity, opportunities exist to leverage conferences, scientific meetings, and other professional spaces to learn from each other and create mentorship opportunities for junior scholars and those who may be moving into this area. This would facilitate continued discussion among practitioners and researchers to create common definitions and goals around HL, DHL, and their relationship to SDoH.

## Abbreviations

DHL: digital health literacy
eHL: electronic health literacy
HL: health literacy
LGBTQIA+: lesbian, gay, bisexual, transgender, queer, questioning, intersex, asexual, and other
NIH: National Institutes of Health
PI: project investigator
RePORTER: RePORT Expenditures and Results
SDoH: social determinants of health
